# Pyroptosis in glioblastoma: A crucial regulator of the tumour immune microenvironment and a predictor of prognosis

**DOI:** 10.1111/jcmm.17200

**Published:** 2022-01-26

**Authors:** Jinhu Lin, Xiang Lai, Xiaoping Liu, Hua Yan, Changwu Wu

**Affiliations:** ^1^ Department of Neurosurgery Meizhou People's Hospital (Huangtang Hospital) Meizhou China; ^2^ Institute of Anatomy University of Leipzig Leipzig Germany

**Keywords:** glioblastoma, prognosis, pyroptosis, risk model, tumour immune microenvironment

## Abstract

Recent studies have shown that pyroptosis, an inflammatory form of cell death, has a dual role in tumorigenesis and tumour progression and affects the prognosis of patients; however, the role of pyroptosis in glioblastoma (GBM) is still unclear. In this study, based on GBM patients' data from two independent cohorts, we performed a comprehensive analysis of the expression and prognostic value of 33 pyroptosis‐associated genes (PAGs) in GBM, as well as their role in the tumour immune microenvironment (TIME) of GBM. We identified 29 PAGs that were differentially expressed between GBM and normal brain tissue, 18 of which were upregulated in GBM tissue. Most of the 33 PAGs were strongly correlated with the levels of immune cell infiltration. Based on the 33 PAGs, the GBM samples can be divided into two clusters (C1‐C2), with C1 having a ‘hot’ but immunosuppressive TIME and C2 having a ‘cold’ TIME, suggesting different immunotherapeutic responses in the two clusters. In addition, we identified four PAGs that were strongly associated with GBM prognosis and constructed a risk model based on these four PAGs. This risk model is an independent prognostic factor for GBM patients, and there is a different immune status between high‐ and low‐risk groups. In conclusion, this study demonstrates that pyroptosis is closely associated with the prognosis and TIME of GBM and provides an important basis for further studies on the relationship between pyroptosis and GBM.

## 1 INTRODUCTION

Glioblastoma (GBM), a grade IV glioma, is the most common primary brain tumour in adults. GBM develops more rapidly and has a worse prognosis compared to low‐grade gliomas.^1^, ^2^ Despite the current comprehensive treatment strategy of a combination of surgical resection and chemotherapy/radiotherapy, its median overall survival (OS) is still only 14.6 months.^3^ Given the limitations of available therapies for GBM, there is an urgent need to further clarify the pathogenesis of GBM and develop new treatment targets to improve patient prognosis. Furthermore, although several biomarkers or gene signatures have been shown to have prognostic predictive ability for GBM,^4^, ^5^ they are all still in the pre‐clinical research phase; so, further uncovering of new prognostic gene signatures is equally important.

Pyroptosis is a form of programmed cell death triggered by inflammatory vesicles, which is manifested by continuous cell distension until the cell membrane ruptures, resulting in the release of cell contents and thus a strong inflammatory response.^6^ Gasdermin (GSDM) proteins are key effector molecules for pyroptosis. This family includes proteins named GSDMA to GSDME and pejvakin (DFNB59 or PJVK).^7^ Although pyroptosis was initially found to be a pivotal mechanism in the fight against infections, more and more studies have also identified it as having an important role in tumour development.^6^ The role of pyroptosis in tumours is complex, and while it has been shown to inhibit tumour growth by enhancing cytotoxic lymphocyte responses,^8^ it has also been reported to accelerate tumour growth by affecting the tumour microenvironment.^9^ For example, the pyroptosis genes GSDMA, GSDMC and GSDMD have all been reported to inhibit gastric cancer cell proliferation,^10^ while GSDMB is amplified and highly expressed in liver, colon and HER2^+^ breast cancers and may play an oncogenic role in these cancers.^11^ In addition, GSDMC has also been reported to be upregulated in colorectal cancer, melanoma and lung adenocarcinoma and promotes tumour growth and metastasis.^12^, ^13^, ^14^ This further responds to the complex role of pyroptosis and its associated genes in tumour development and anti‐tumour immunity and the potential tumour type specificity. Several other studies have shown that pyroptosis is closely related to the regulation of the tumour immune microenvironment (TIME), where GSDMD was found to be required for cytotoxic T lymphocytes to exert anti‐tumour responses in lung cancer,^15^ and it was also found that pyroptosis plays an important role in the anti‐tumour response of natural killer cells.^16^

Based on the above background, pyroptosis is known to play a key role in the development and anti‐tumour immunity of a variety of tumours, yet little is known about pyroptosis in GBM. The aim of this study was to investigate the expression profile and prognostic value of pyroptosis‐associated genes (PAGs) in GBM and to reveal the correlations between them and the TIME of GBM. This contributes to further understanding the epigenetic changes that occur during GBM tumorigenesis and identifying potential prognostic biomarkers and therapeutic targets.

## 2 MATERIALS AND METHODS

### 2.1 Datasets

RNA sequencing (RNA‐seq) data and clinical information for 143 GBM samples from The Cancer Genome Atlas (TCGA) cohort and RNA‐seq data for 196 normal human brain samples in the Genotype‐Tissue Expression (GTEx) cohort were obtained from the Xena Functional Genomics Explorer (https://xenabrowser.net/datapages/) (Table S1). RNA‐seq data were normalized by log2(TPM+1) transformation to make the two cohorts comparable. The normalized expression matrix of 119 GBM samples in the Repository of Molecular Brain Neoplasia Data (REMBRANDT) cohort was obtained from GlioVis (http://gliovis.bioinfo.cnio.es/)^17^ and clinical information was obtained from the Chinese Glioma Genome Atlas. Samples without complete clinical information were excluded from the study. The VarScan‐processed mutation dataset in the TCGA cohort was obtained from the Genomic Data Commons Data Portal (https://portal.gdc.cancer.gov/).

### 2.2 Identification of differentially expressed PAGs

A total of 33 PAGs were first extracted by searching the existing literature (Table S2).^9^, ^18^, ^19^ This was followed by the identification of differentially expressed PAGs among GBM tumour tissues from TCGA cohort and normal brain tissues from the GTEx cohort using the R package ‘limma’. The STRING tool (https://string‐db.org/) was used to further construct a protein–protein interaction (PPI) network with a minimum required interaction score of 0.9 for differentially expressed PAGs,^20^ and the hub genes of this PPI network were identified in Cytoscape software using the cytoHubba plugin.^21^ Statistical significance was set at *p* < 0.05.

### 2.3 Correlation analysis and mutation analysis of PAGS

Correlations among the 33 PAGs were analysed using Pearson's correlation analysis and visualized using a heatmap. The waterfall plot of mutation frequency for the 33 PAGs in TCGA cohort was generated using the R package ‘maftools’. Tumour mutation burden (TMB) was also calculated using the R package ‘maftools’. Statistical significance was set at *p* < 0.05.

### 2.4 Analysis of the role of PAGs in the TIME of GBM

The immune, stromal and ESTIMATE scores were calculated for each sample using the R package ‘ESTIMATE’, and correlations with the 33 PAGs were calculated using Pearson's analysis. The single‐sample gene set enrichment analysis (ssGSEA) was performed using the R package ‘GSVR’ to quantify the infiltration score of the 28 previously reported immune cells in each sample.^22^ Statistical significance was set at *p* < 0.05.

### 2.5 Identification of potential pyroptosis‐related subgroups

Based on the expression of pyroptosis‐related genes, the partition around medoids algorithm was used to perform unsupervised consensus clustering to identify potential pyroptosis‐related clusters in the TCGA and REMBRANDT cohorts. A total of 1000 bootstraps were performed, and each bootstrap included 80% of the patients, with the number of clusters set at 2–10. In addition, the intra‐group proportion (IGP) analysis was performed to assess the reproducibility and similarity of clusters between the TCGA and REMBRANDT cohorts.^23^ The Wilcox test and *t*‐test were used to compare the level of immune infiltration between the two clusters, and gene set enrichment analysis (GSEA) was used to perform functional enrichment analysis of the different clusters.^24^

### 2.6 Establishment and validation of the PAG prognostic signature

Univariate Cox regression analysis was performed using the R package "survival" to assess the prognostic value of each PAG in the TCGA cohort; the cut‐off *p*‐value was set to 0.1 to prevent omission and a total of five PAGs that were strongly associated with survival in GBM were identified. Then, the R packages ‘glmnet’ and ‘survival’ were used to perform LASSO Cox regression analysis to narrow down the candidate PAGs. Multivariate Cox regression analysis was used to calculate the correlation coefficients and establish a risk model. In addition, the ‘survminer’ R package was used to find the best cut‐off value to classify samples into high‐ and low‐risk groups. The Kaplan–Meier curve was used to estimate the difference in OS between the two groups, and a log‐rank test was used to evaluate its statistical significance. The R package ‘timeROC’ was used to plot the receiver operating characteristic (ROC) curve over time to assess the predictive value of this prognostic gene signature. A risk model based on the prognostic gene signature was also established and validated in the REMBRANDT cohort. Further, univariate and multivariate regression analyses were performed to determine the independent prognostic value of the prognostic gene signature, using age and gender as covariates. Statistical significance was set at *p* < 0.05.

### 2.7 Enrichment analysis of differential genes (DEGs) between the low‐ and high‐risk groups

The R package ‘limma’ was used to filter DEGs between low‐ and high‐risk groups in the TCGA cohort based on the thresholds |log_2_FC| ≥1 and FDR <0.05. Based on these DEGs, the R package ‘clusterProfiler’ was used to perform Gene ontology (GO) enrichment analysis and Kyoto Encyclopedia of Genes and Genomes (KEGG) pathway analysis. Statistical significance was set at *p* < 0.05.

## 3 RESULTS

### 3.1 Expression and mutation profiles of PAGs in GBM

**FIGURE 1:**
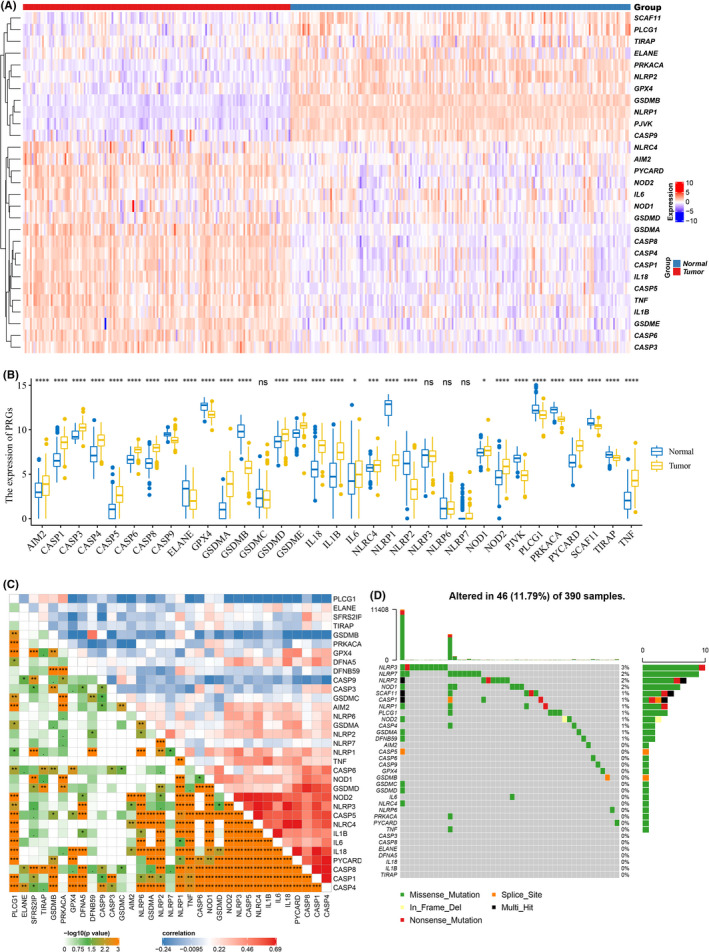
Expression and mutation of 33 pyroptosis‐associated genes. (A) Heatmap (blue: low expression level; red: high expression level) of the pyroptosis‐associated genes between normal and tumour tissues. (B) Box plots of expression differences between normal and tumour tissues (blue: normal; yellow: tumour). (C) Correlations among the 33 pyroptosis‐associated genes using Pearson's analysis. Negative correlation was marked with blue and positive correlation with red. (D) Waterfall plot of the mutation profiles of the 33 pyroptosis‐associated genes. *p* <  0.1, *p < 0.05, **p  < 0.01, ***p < 0.001, ****p  < 0.0001

By comparing the expression levels of 33 PAGs in 143 GBM tumour tissues from the TCGA cohort and 196 normal brain tissues from the GTEx cohort, it was found that most of the PAGs were differentially expressed (Figure 1A). Specifically, there were 29 differentially expressed PAGs, of which 18 were upregulated (AIM2, CASP1, CASP3, CASP4, CASP5, CASP6, CASP8, GSDMA, GSDMD, GSDME, IL18, IL1B, IL6, NLRC4, NOD1, NOD2, PYCARD and TNF) and 11 were downregulated (CASP9, ELANE, GPX4, GSDMB, NLRP1, NLRP2, PJVK, PLCG1, PRKACA, SCAF11 and TIRAP) in tumour tissues (Figure 1B). To further explore the interactions of these differentially expressed PAGs, we constructed a PPI network and identified CASP1, PYCARD, NLRP1, AIM2 and NLRC4 as the top5 hub genes (Figure S1). In addition, correlation analysis revealed substantial co‐expression among 33 PAGs, with CASP4 expression being positively correlated with the expression of most PAGs, while PLCG1 expression was negatively correlated with the expression of most PAGs (Figure 1C). Genetic variation analysis in GBM showed that mutations in PAGs occurred in only 11.79% of the cases, and most of them were missense mutations, with NLRP3 having the highest mutation frequency (Figure 1D).

### 3.2 The role of PAGs in the TIME of GBM

To explore the role of PAGs in the TIME of GBM, the correlation of PAGs with stromal, immune and ESTIMATE scores, where the ESTIMATE score represents tumour purity, was first assessed in the TCGA cohort. Most PAGs were found to have significant positive correlations with stromal, immune and ESTIMATE scores, while CASP9, GSDMB and PLCG1 were negatively correlated with these scores (Figure S2A). In addition, the correlations between the 33 PAGs and the infiltration of 28 immune cells were assessed. Not surprisingly, most PAGs were positively correlated with immune cell infiltration, whereas CASP9, GSDMB and PLCG1 were negatively correlated with it (Figure S2B). Notably, almost all PAGs were negatively correlated with type‐2 T helper cell infiltration. These results imply that PAGs may be involved in the regulation of TIME of GBM.

### 3.3 GBM subgroups with specific expression patterns of PAGs exhibit different immune signatures

**FIGURE 2:**
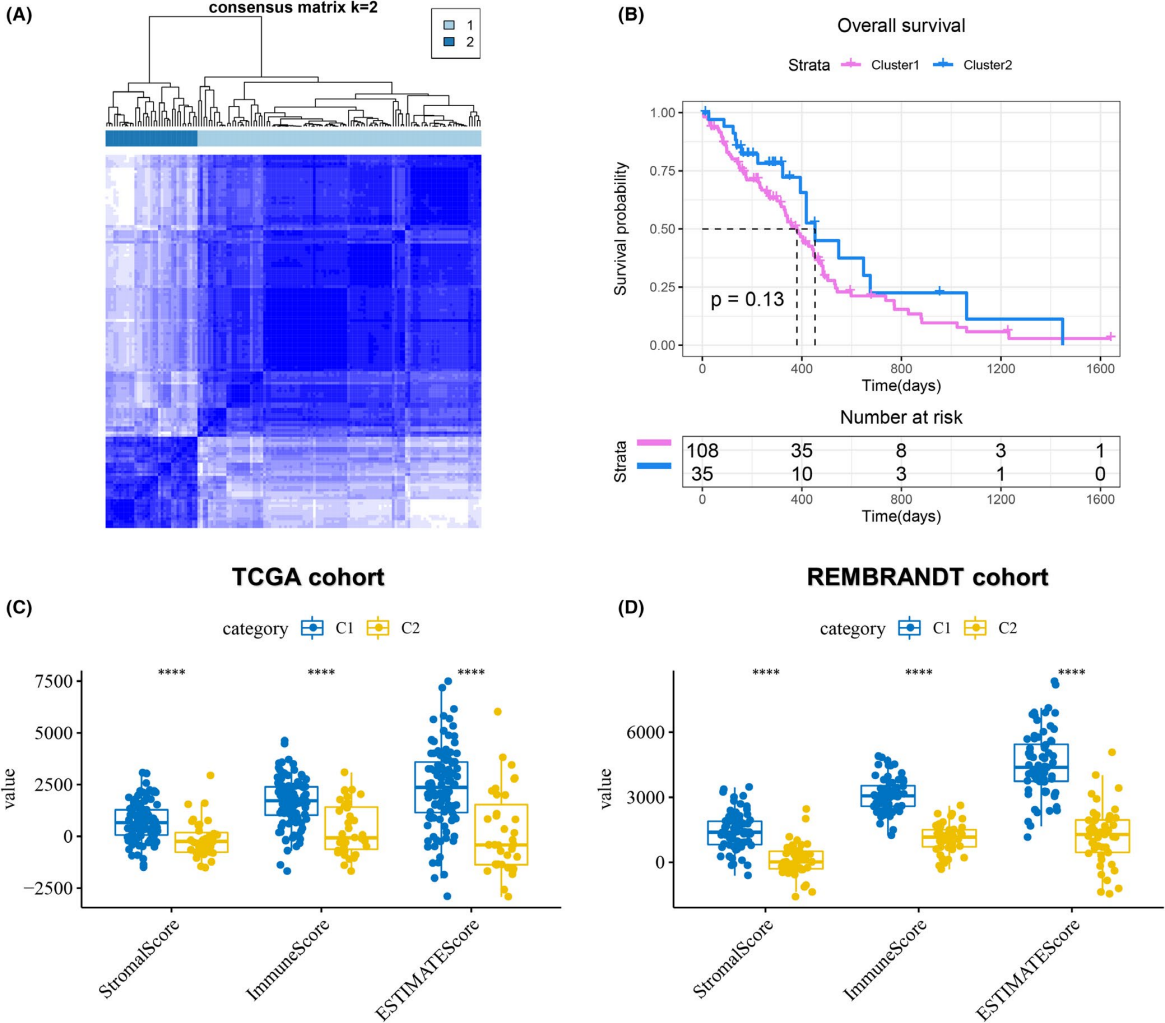
Tumour classification based on 33 pyroptosis‐associated genes. (A) 143 GBM patients from the TCGA cohort were grouped into two clusters according to the consensus clustering matrix (*k* = 2). (B) Kaplan–Meier curve of OS for the two clusters. (C) Differences in stromal, immune and ESTIMATE scores between the two clusters (blue: cluster 1; yellow: cluster 2) in the TCGA cohort. (D) Differences in stromal, immune and ESTIMATE scores between the two clusters (blue: cluster 1; yellow: cluster 2) in the REMBRANDT cohort. ****p  < 0.0001. GBM, Glioblastoma

To explore the association between 33 PAGs and potential subgroups of GBM, unsupervised consensus clustering was performed based on the expression profile of PAGs in the TCGA cohort and the K value was set to 2 according to the cumulative distribution function. It was found that GBM samples could be classified into two clusters, namely cluster 1 (C1) and cluster 2 (C2) (Figure 2A). No significant differences were found when comparing the OS between C1 and C2 (*p* = 0.13, Figure 2B). Similarly, the GBM samples were divided into C1 and C2 in the REMBRANDT cohort (Figure S3A), and there was no significant difference in the OS between the two groups (*p* = 0.59, Figure S3B). Subsequently, the IGP analysis further determined the consistency and reproducibility of the subgroups in the two cohorts (Table S3).

**FIGURE 3:**
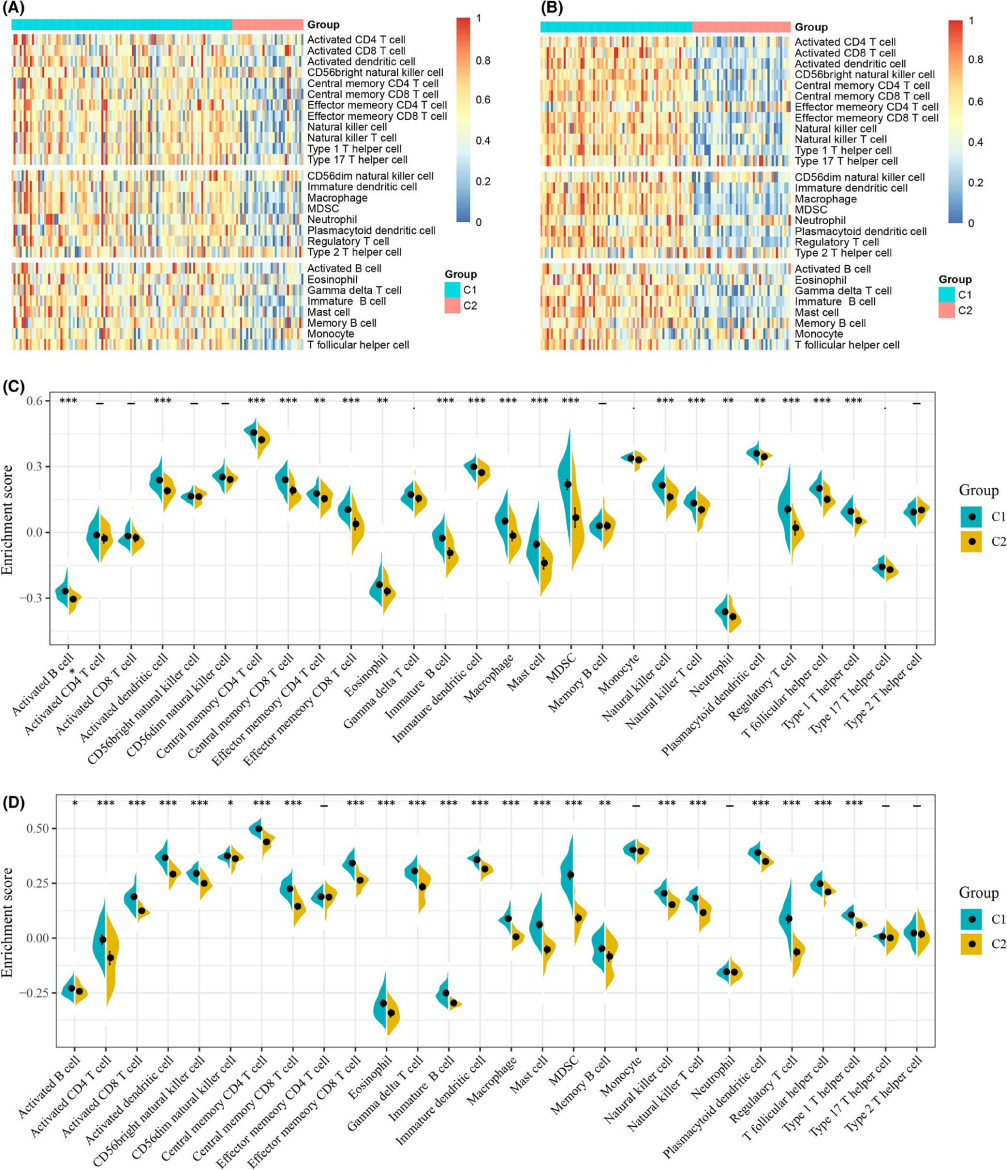
Cellular characteristic of the two clusters in GBM. (A,B) Heatmaps of 28 previously reported immune cell signatures scores between the two GBM clusters in the TCGA cohort (A) and the REMBRANDT cohort (B). (C,D) Differences of 28 immune cell signatures scores between the two GBM clusters in the TCGA cohort (C) and the REMBRANDT cohort (D). – *p* ≥ 0.1, *p* < 0.1, *p < 0.05, **p < 0.01, ***p < 0.001. GBM, Glioblastoma

In addition, C1 had higher stromal, immune and ESTIMATE scores in both the TCGA and REMBRANDT cohorts (Figure 2C,D). Further, we explored the infiltration of 28 immune cells into different clusters. In the TCGA cohort, C1 and C2 had distinctly different levels of immune cell infiltration (Figure 3A). As shown in Figure 3C, C1 had not only a higher infiltration of immunostimulatory cells such as activated dendritic cells and natural killer cells, but also a higher infiltration of immunosuppressive cells such as myeloid‐derived suppressor cells (MDSCs) and regulatory T cells compared with C2. Therefore, C1 is an immunologically hot but immunosuppressive phenotype, whereas C2 is an immunologically cold phenotype. Importantly, similar trends were also observed in the REMBRANDT cohort (Figure 3B,D). In addition, GSEA in the TCGA cohort also showed that C1 was mainly enriched in B‐cell receptor signalling pathway, chemokine signalling pathway, while C2 was mainly enriched in cell cycle and DNA replication. (Figure S4). Further, we calculated the TMB in each patient using the VarScan‐processed mutation dataset of TCGA. TMB analysis of C1 and C2 showed that there was no significant difference in TMB between the two clusters (Figure S5).

### 3.4 Establishment and validation of the pyroptosis‐related prognostic gene model

**FIGURE 4:**
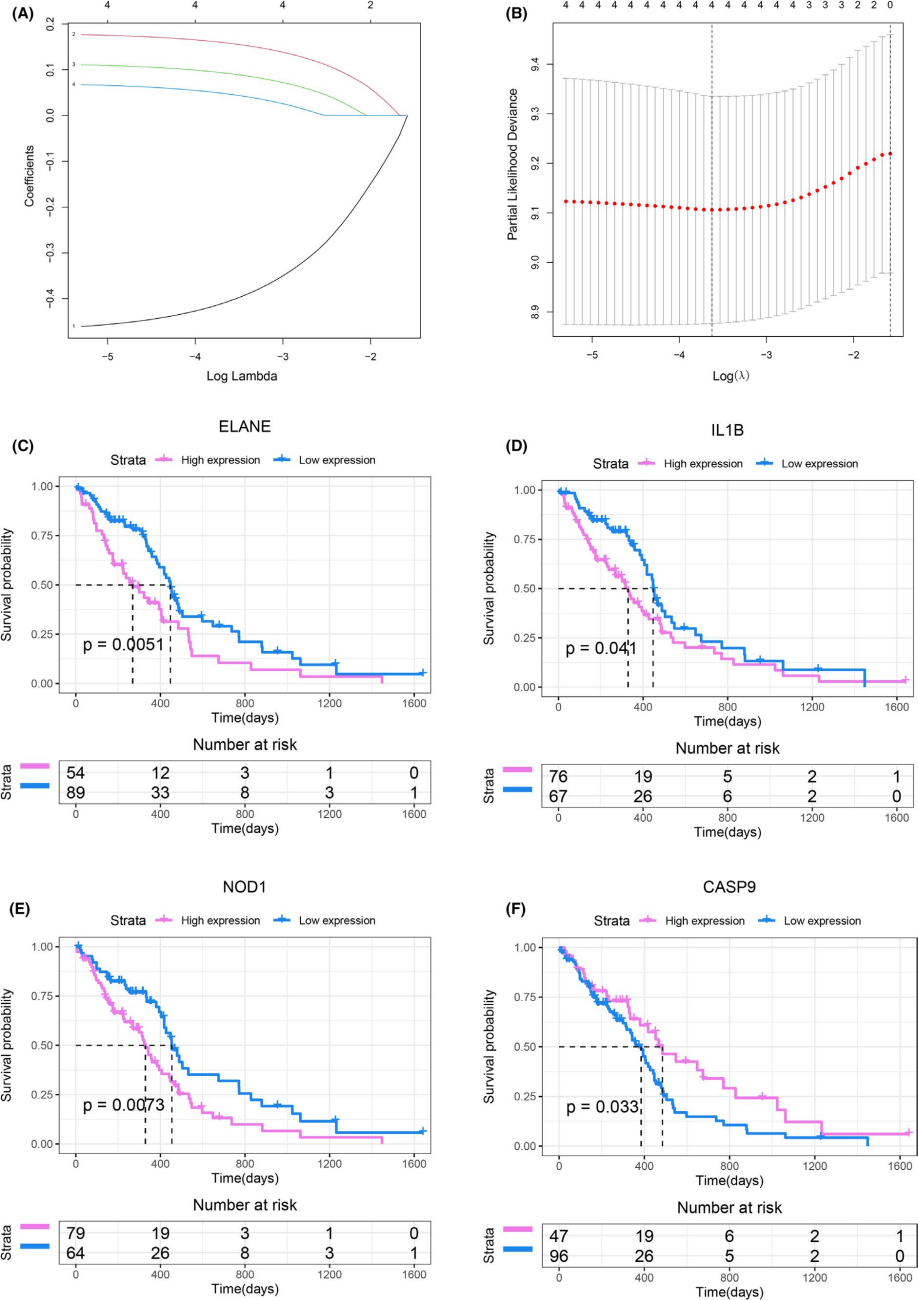
Construction of risk model in the TCGA cohort. (A) LASSO coefficient profiles of the four OS‐related genes. (B) Cross‐validation for tuning the parameter selection in the LASSO regression. (C–F) The OS Kaplan–Meier curves of ELANE (C), IL1B (D), NOD1 (E) and CASP9 (F) in the high‐/low‐expression group. OS, Overall survival

To construct a prognostic gene model, the prognostic value of 33 PAGs was first assessed in the TCGA cohort using univariate Cox regression (Table S4), and a total of 5 PAGs closely associated with GBM OS were screened. Subsequently, LASSO regression analysis was performed to further narrow down and screen 4 candidate genes, namely ELANE, IL1B, NOD1 and CASP9 (Figure 4A,B). All four genes were associated with the prognosis of GBM, as shown in Figure 4C–F. GBM patients with high expression of ELANE (*p* = 0.0051), IL1B (*p* = 0.041) and NOD1 (*p* = 0.0073) had poorer OS, while those with high expression of CASP9 (*p* = 0.033) had better OS. Finally, multivariate Cox regression analysis was performed on these four PAGs to construct a risk model. The risk score for each sample was calculated as follows:

$$\text{risk score}\;=\left(0.18133*\text{ELANE}\;\exp.\right)+\left(0.11453*\text{IL1B}\;\exp.\right)+\left(0.07369*\;\text{NOD1}\;\exp.\right)+\left(‐0.47153*\text{CASP9}\;\exp.\right)$$

**FIGURE 5:**
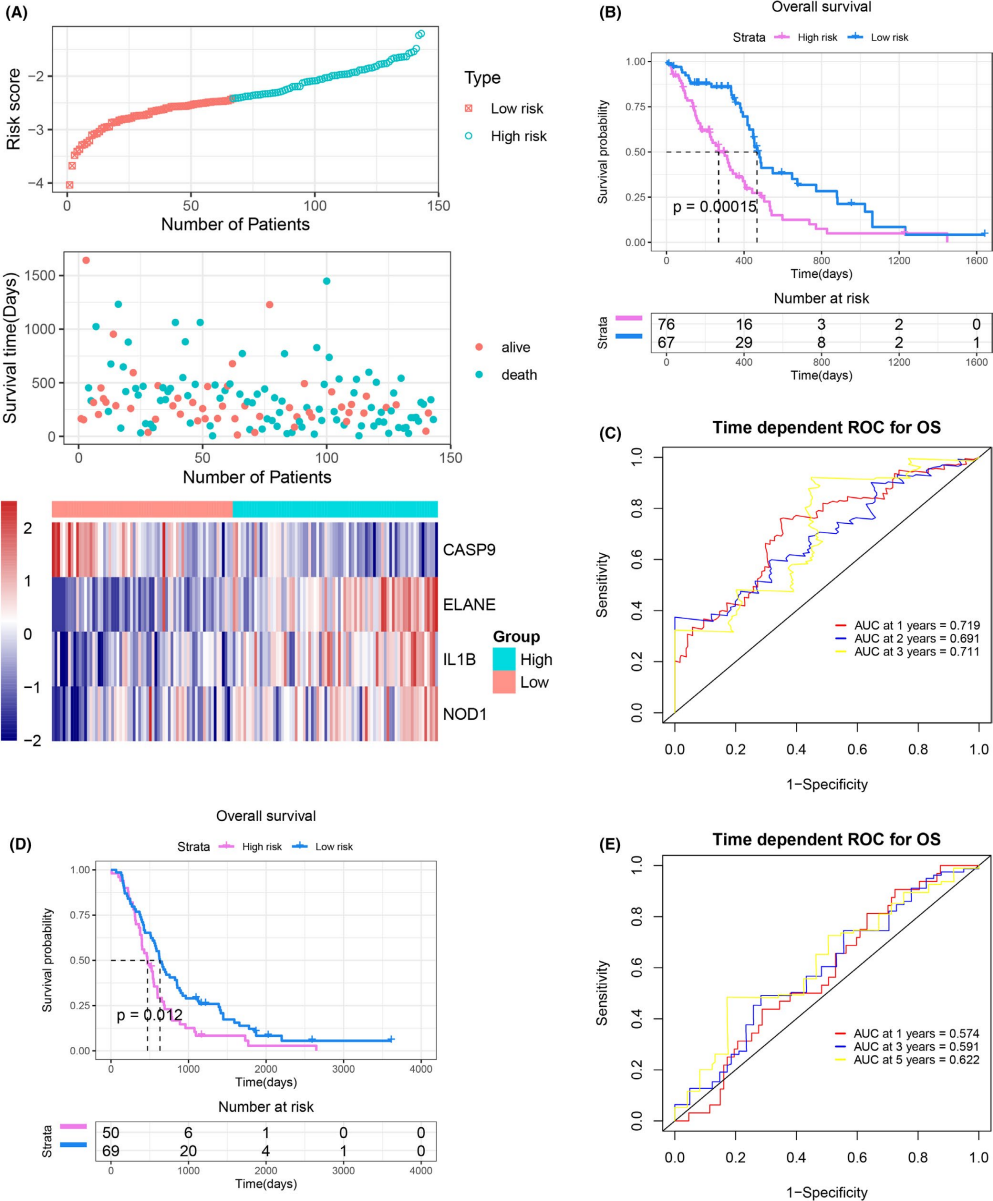
Prognostic value of the risk model. (A) Distribution of risk score, survival status and the expression of four prognostic pyroptosis‐associated genes in the TCGA cohort. (B) The Kaplan–Meier curve for the OS of patients in the high‐ and low‐risk groups in the TCGA cohort. (C) The ROC curve demonstrated the predictive efficiency of the risk score in the TCGA cohort. (D) The Kaplan–Meier curve for the OS of patients in the high‐ and low‐risk groups in the REMBRANDT cohort. (E) The ROC curve demonstrated the predictive efficiency of the risk score in the REMBRANDT cohort. OS, Overall survival

GBM patients in the TCGA cohort were divided into high‐ and low‐risk groups based on the best cut‐off value of the risk score, and risk scores were calculated according to the risk model. Patients were also grouped in the REMBRANDT cohort. In the TCGA cohort, the high‐risk group had more deaths and shorter survival time, with higher expression of ELANE, IL8B and NOD1 and lower expression of CASP9 compared with patients in the low‐risk group (Figure 5A). Similar trends were observed in the REMBRANDT cohort (Figure S6). Kaplan–Meier curves showed that GBM patients in the high‐risk group had significantly shorter OS than those in the low‐risk group in the TCGA cohort (*p* = 0.00015, Figure 5B). In addition, the ROC curve showed that the areas under the curves (AUC) for 1‐, 2‐ and 3‐year OS were 0.719, 0.691 and 0.711 respectively (Figure 5C). In the REMBRANDT cohort, there was also a significant difference in OS between the high‐ and low‐risk groups (Figure 5D), and the AUCs of 1‐, 2‐ and 3‐year OS were 0.574, 0.59, and 0.622, respectively (Figure 5E), which indicated a good predictive efficacy of our model.

### 3.5 Independent prognostic value of our risk model

To verify the independent prognostic effect of the pyroptosis‐related prognostic gene model, univariate and multivariate Cox regression analyses were performed. In the TCGA cohort, risk score was confirmed as an independent prognostic factor for OS in GBM patients in both univariate (hazards ratio [HR] = 2.718, 95% confidence interval [CI]: 1.708–4.325) and multivariate (HR = 2.436, 95% CI: 1.502–3.949) Cox regression analyses (Table 1). In the REMBRANDT cohort, it was also found that the risk score was an independent prognostic factor for OS in both univariate (HR = 1.555, 95% CI: 1.061–2.278) and multivariate (HR = 1.719, 95% CI: 1.161–2.546) Cox regression analyses (Table 2).

**TABLE 1:** Univariate and multivariate analysis of the prognostic value of the four‐gene signature in terms of OS in the TCGA cohort

Characteristics	Univariate analysis			Multivariate analysis		
	HR	95% CI	*p*‐value	HR	95% CI	*p*‐value
Age	1.031	1.011~1.051	0.002	1.024	1.004~1.046	0.022
Gender (male vs. female)	0.782	0.513~1.191	0.252	0.845	0.552~1.292	0.436
Risk score	2.718	1.708~4.325	<0.001	2.436	1.502~3.949	<0.001

**TABLE 2:** Univariate and multivariate analysis of the prognostic value of the four‐gene signature in terms of OS in the REMBRANDT cohort

Characteristics	Univariate analysis			Multivariate analysis		
	HR	95% CI	*p*‐value	HR	95% CI	*p*‐value
Age	1.041	1.023~1.059	<0.001	1.043	1.024~1.061	<0.001
Gender (male vs. female)	1.123	0.763~1.653	0.555	1.230	0.828~1.827	0.304
Risk score	1.555	1.061~2.278	0.023	1.719	1.161~2.546	0.006

### 3.6 Functional analyses based on the pyroptosis‐related prognostic gene signature

**FIGURE 6:**
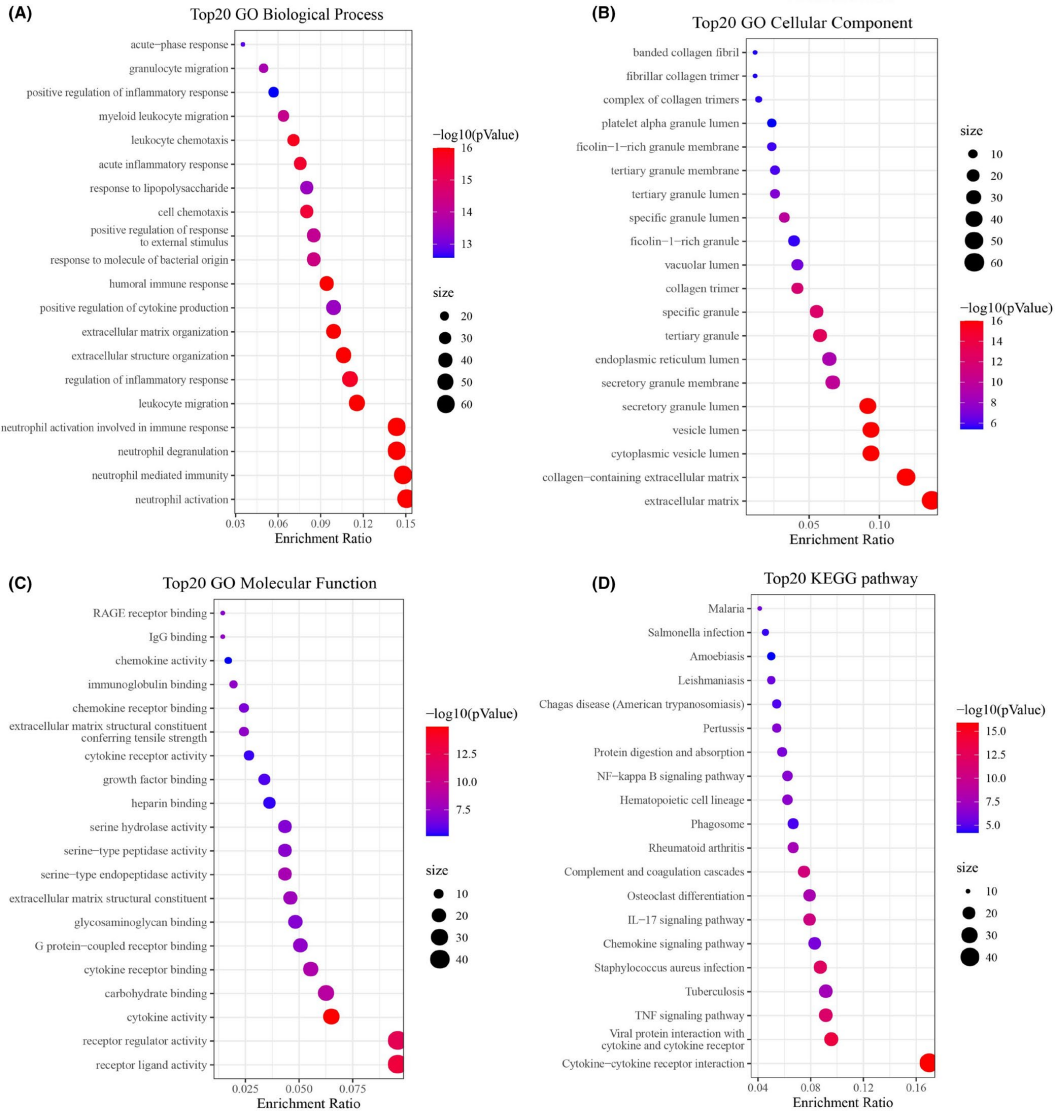
Functional analysis based on the DEGs between the two‐risk groups in the TCGA cohort. (A–C) The top 20 enriched items in gene ontology analysis, including (A) biological process, (B) cellular component and (C) molecular function. (D) The top 20 enriched items in Kyoto Encyclopedia of Genes and Genomes analysis. The size of circles represented the number of genes enriched

To further explore the differences in pathways and gene function between the high‐risk and low‐risk groups, 501 DEGs were extracted with the thresholds |log_2_FC| ≥1 and FDR <0.05, of which 378 DEGs were upregulated and 123 DEGs were downregulated in the high‐risk group. Further, the GO enrichment analysis indicated that these DEGs were mainly related to biological processes such as ‘leukocyte migration’ and ‘regulation of inflammatory response’ (Figure 6A), cellular components such as ‘extracellular matrix’ and ‘collagen−containing extracellular matrix’ (Figure 6B) and molecular functions such as ‘receptor regulator activity’ and ‘cytokine activity” (Figure 6C). KEGG pathway analysis indicated that these DEGs may be involved in multiple immune functions through the ‘cytokine−cytokine receptor interaction’, ‘chemokine signalling pathway’ and ‘IL‐17 signalling pathway’ (Figure 6D). These results suggest that these DEGs may be closely related to multiple immune responses and immune processes.

### 3.7 Comparison of the immune activity between high‐risk and low‐risk groups

**FIGURE 7:**
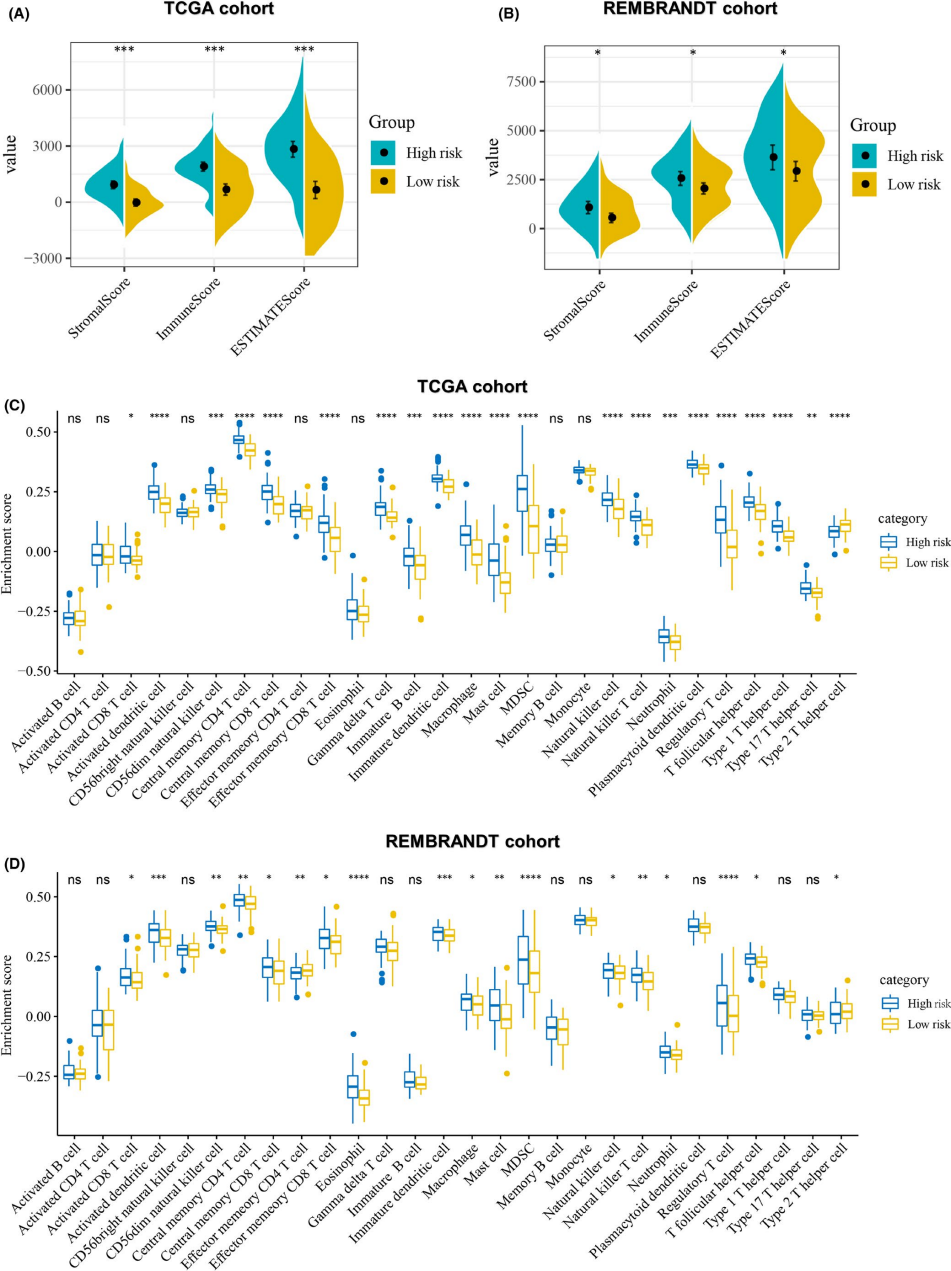
Immune characteristics of the high‐ and low‐risk groups. (A) Differences in stromal, immune and ESTIMATE scores between the high‐ and low‐risk groups in the TCGA cohort. (B) Differences in stromal, immune and ESTIMATE scores between the high‐ and low‐risk groups in the REMBRANDT cohort. (C) Differences of 28 immune cell signatures scores between the high‐ and low‐risk groups in the TCGA cohort. (D) Differences of 28 immune cell signatures scores between the high‐ and low‐risk groups in the REMBRANDT cohort. *p < 0.05, **p < 0.01, ***p < 0.001

We compared the differences between stromal, immune and ESTIMATE scores and levels of immune cell infiltration between the high‐ and low‐risk groups in TCGA and REMBRANDT cohorts. As shown in Figure 7A,B, the high‐risk group in both cohorts had higher stromal, immune and ESTIMATE scores than the low‐risk group. In the TCGA cohort, the high‐risk group generally had higher levels of immune cell infiltration compared with the low‐risk group, including multiple immunostimulatory cells (such as activated CD8^+^T cells, activated dendritic cells and natural killer cells) and immunosuppressive cells (such as MDSCs, immature dendritic cells and regulatory T cells) (Figure 7C). Similar results were observed in the REMBRANDT cohort (Figure 7D).

### 3.8 The relationship between the risk model and TMB

In the TCGA cohort, we found that the low‐risk group had a relatively higher TMB than the high‐risk group, although this result was not statistically significant (*p* = 0.077, Figure S7A). In the study of the correlation of four genes in the risk model with TMB, we found that the expression levels of IL8B (*p* = 0.041, *r* = −0.17) and NOD1 (*p* = 0.024, *r* = −0.19) were negatively correlated with TMB levels (Figure S7C,D), while no significant correlation was found for ELANE and CASP9 (Figure S7B,E).

## 4 DISCUSSION

Pyroptosis, an inflammatory form of cell death that activates the immune system, has recently attracted increasing attention and interest from oncologists. Recent studies have shown that pyroptosis has a dual role as a ‘double‐edged sword’ in the development and treatment of tumours.^9^, ^25^ On the one hand, normal cells may transform into tumour cells after being stimulated by the large amount of inflammatory factors released during pyroptosis.^18^ On the other hand, tumour‐cell death induced by pyroptosis pathway makes it a new potential target for tumour therapy.^9^, ^26^ In fact, in a recent study, it was found that anti‐tumour immune responses induced by pyroptosis gene “GSDMA3” led to significant tumour shrinkage in two mouse models of breast cancer, which was associated with the increase of pyroptosis cells and depended on the increased T‐cell and NK‐cell infiltration.^27^ This indicated the great potential of pyroptosis in tumour therapy and the close link to the immune response. Interestingly, this study also found that GSDMA3 significantly enhanced the response to anti‐PD1 therapy, suggesting that the inflammatory effects activated by GSDMs improved the efficacy of immunotherapy. This was particularly achieved by increasing lymphocyte infiltration, namely turning tumours from ‘cold’ to ‘hot’, which indicated the key role of pyroptosis in tumour TIME.^8^, ^27^ However, the role of pyroptosis and PAGs in GBM has not been elucidated yet.

In the present study, we explored, for the first time, the expression of PAGs, their relationship with TIME, and their prognostic value in GBM. Compared with normal brain tissue, 29 of 33 PAGs were dysregulated in GBM tissues, indicating the possible important role of PAGs in GBM tumorigenesis. Furthermore, the strong correlations between PAGs and immune score, stromal score and immune cell infiltration also implied that pyroptosis could be involved in the regulation of TIME in GBM. Consensus clustering based on 33 PAGs identified two GBM subgroups with significantly different immune characteristics, indicating completely different responses to immunotherapy. C1 tumours were identified as having a hot but immunosuppressive phenotype, characterized by higher immune scores, stromal scores and immune cell infiltration. Therefore, the use of immune checkpoint blockers (ICBs) in C1 tumours to augment pre‐existing anti‐tumour immunity may be a promising strategy. As previous studies have shown that GBM patients struggle to benefit from monotherapy with ICB,^28^, ^29^ the use of ICB multidrug combinations or ICB monotherapy in combination with other emerging therapies such as chimeric antigen receptor T‐cell therapy^30^ and potential pyroptosis‐targeted therapy could be more effective. C2 tumours were identified as having cold phenotype characterized by low immune cell infiltration and low immune and stromal scores. Thus, pyroptosis‐targeted therapy or tumour vaccine therapy may be able to increase lymphocyte infiltration and activate the anti‐tumour inflammatory response, thereby turning C1 tumours from ‘cold’ to ‘hot’. This suggests that our classification approach based on PAGs may be beneficial for the selection of immunotherapeutic strategies and future pyroptosis‐targeted therapies. In addition, in previous studies, benzimidazole was found to trigger GBM pyroptosis via the NF‐κB/NLRP3/GSDMD pathway to achieve anti‐tumour effects.^31^ Galangin can also achieve anti‐tumour effects in GBM by inducing pyroptosis.^32^ This demonstrates the great potential of pyroptosis‐targeted therapy in combination with chemotherapy in GBM.

In this study, we identified four PAGs (ELANE, IL1B, NOD1 and CASP9) that were strongly associated with GBM prognosis and constructed a prognostic gene signature based on them. ELANE is a serine protease secreted by neutrophils, and a recent study showed that ELANE selectively killed tumour cells and attenuated tumour growth,^33^ while a study by Deryugina et al. showed that ELANE promoted tumour cell intravasation and early metastasis.^34^ Ye et al. also identified ELANE as a risk factor for poor prognosis in ovarian cancer.^19^ In our study, high expression of ELANE was also associated with poor prognosis in GBM. These results seem contradictory, suggesting that the role of ELANE in tumours could be tumour type‐specific and that the role of ELANE in GBM warrants further study and confirmation. IL1B, a potent pro‐inflammatory cytokine, was initially found to be a major endogenous pyrogen, with close association with T cell, neutrophil and B cell activation and cytokine production.^35^ In the pyroptosis process, activated caspase‐1 cleaves IL1B precursors to form activated IL1B, which recruits inflammatory cells extracellularly, amplifies the inflammatory response and is an important pyroptosis effector.^6^ However, IL1B was found to be upregulated in a variety of tumours and related to poor prognosis.^36^ Together with vascular endothelial growth factor, IL1B establishes and maintains tumour‐mediated angiogenesis,^37^ while IL1B‐knockout mice tumour model showed significant tumour reduction.^38^ Chronic inflammation is widely recognized as one of the hallmarks of carcinogenesis, tumour progression and metastasis,^39^ and IL1B exerts its oncogenic effects by promoting chronic inflammation. In addition, IL1B has been found to be an important stimulus for the expansion and migration of MDSCs,^40^ promoting immunosuppression and downregulating immune surveillance through multiple mechanisms.^41^ These results are consistent with our results and provide a theoretical basis for IL1B as a poor prognostic factor in GBM. In fact, interception of IL‐1β significantly reduced the incidence of lung cancer in a clinical study,^42^ suggesting the great potential of IL1B as a new therapeutic target for GBM. NOD1 is a member of the NOD family that can play a role in innate immunity by acting as a pattern recognition receptor that binds to bacterial peptidoglycan and triggers inflammation.^43^ A recent study has shown that NOD1 promotes immunosuppression and regulates pro‐tumour TIME by regulating myeloid cells stimulating MDSC amplification, and maintaining its immunosuppressive potential.^44^ Consistent with our results, NOD1 was also identified as a poor prognostic factor for lung adenocarcinoma in a study by Lin et al.^45^ CASP9 is a member of the caspase family widely believed to play a central role in apoptosis and is a tumour suppressor.^46^ In addition, CASP9 also contributes to necroptosis, a form of immune system‐mediated cell death.^46^ In GBM, a recent study about the mechanism of the first‐line chemotherapeutic agent temozolomide suggests that it promotes apoptosis in GBM cells by upregulating cation‐transport regulatory protein 1 to activate CASP3/9.^47^ In our study, CASP9 was also found to be negatively associated with stromal score and the infiltration of immunosuppressive cells such as MDSCs in GBM, implying that CASP9 may also exert anti‐tumour effects by regulating the TIME of GBM. Therefore, targeting CASP9, and thereby promoting apoptosis and regulating TIME, may have a potential therapeutic role. Overall, our study constructed a risk model with these four PAGs and validated it in an independent cohort, which provides more choices for prognostic prediction in GBM. However, the processes and mechanisms of their involvement in GBM pyroptosis remain unknown and need to be further investigated.

In the present study, we also investigated the function of DEGs between high‐ and low‐risk groups and found that they are mainly involved in the regulation of immune and inflammatory responses. Moreover, we explored the correlation of PAGs with numerous immune cell infiltrations and GSEA. Based on both of these findings, it is reasonable to speculate that pyroptosis is involved in and regulates the TIME of GBM. Notably, previous studies have shown that pre‐existing anti‐tumour immunity is associated with better prognosis^48^; however, the present study found higher immune cell infiltration in the high‐risk group compared with the low‐risk group. Considering that the high‐risk group not only had higher immunostimulatory cell infiltration, but also significantly higher stromal score and immunosuppressive cell infiltration (such as MDSCs and regulatory T cells), determining the relative advantage of immunostimulatory and immunosuppressive factors in GBM patients is critical for evaluating prognosis. In fact, several studies demonstrate that the immunosuppressive tumour microenvironment is a key factor in the poor prognosis of GBM patients.^48^, ^49^, ^50^, ^51^ The study by Li et al. also found a similar phenomenon in squamous cell carcinoma, which further supports our findings.^52^ Therefore, the worse prognosis in the high‐risk group may be due to intense immunosuppression. Improving the TIME of GBM using a variety of strategies, including targeting pyroptosis, could have important therapeutic implications. As we have previously described, the high‐risk group with high levels of immune infiltration and strong immunosuppression may be better suited for ICB therapy in combination with other treatments including targeted therapies, while the low‐risk group may be better suited for other treatments including tumour vaccines due to the presence of ‘cold’ TME. This is consistent with the view of Huang et al.^53^

This study explored the differential expression of PAGs, their prognostic significance and their role in TIME of GBM. Although this study needs to be further validated in in vivo experiments, it still demonstrated that pyroptosis is closely associated with both prognosis and TIME of GBM and constructed a risk model based on four PAGs that can be used to predict GBM OS. This provides a theoretical basis for further studies on the relationship between pyroptosis and GBM.

## CONFLICT OF INTEREST

The authors declared no potential conflicts of interest.

## AUTHOR CONTRIBUTIONS

**Jinhu Lin:** Conceptualization (equal); Data curation (equal); Formal analysis (equal); Investigation (equal); Methodology (equal); Visualization (equal); Writing – original draft (equal); Writing – review & editing (equal). **Xiang Lai:** Data curation (equal); Formal analysis (equal); Writing – review & editing (equal). **Xiaoping Liu:** Data curation (equal); Formal analysis (equal); Visualization (equal). **Hua Yan:** Formal analysis (equal); Writing – review & editing (equal). **Changwu Wu:** Conceptualization (equal); Data curation (equal); Formal analysis (equal); Investigation (equal); Supervision (lead); Visualization (equal); Writing – original draft (equal); Writing – review & editing (equal).

## Supporting information

Fig S1‐S7

Tab S1

Tab S2

Tab S3

Tab S4

## Data Availability

All data generated and described in this article are available from the corresponding web servers, and are freely available to any scientist wishing to use them for noncommercial purposes, without breaching participant confidentiality. Further information is available from the corresponding author on reasonable request.
