# Supplementary material for: Pyroptosis in glioblastoma: A crucial regulator of the tumour immune microenvironment and a predictor of prognosis
Source: J Cell Mol Med. 2022 Jan 26;26(5):1579–93. doi: 10.1111/jcmm.17200 (PMC8899201; doi:10.1111/jcmm.17200)
Supplement: Supplementary file 1 — Fig S1‐S7 [file JCMM-26-1579-s004.docx]

Supplementary Materials


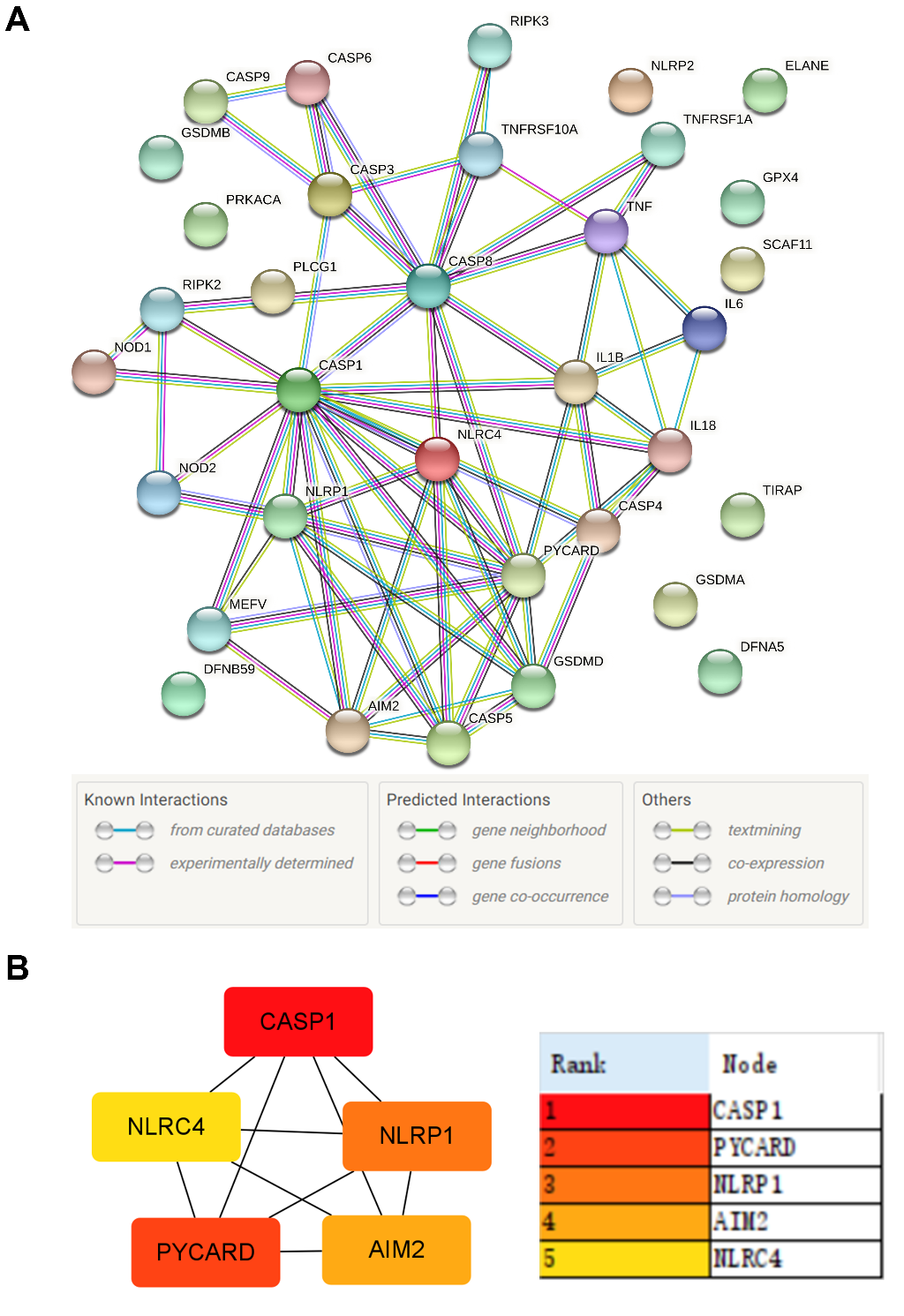


**Supplementary Figure S1.** The interactions among the 33 pyroptosis-associated genes. **(A)** PPI network showing the interactions of the pyroptosis-associated genes. **(B)** Top 5 hub genes in the PPI network. PPI, Protein-protein network


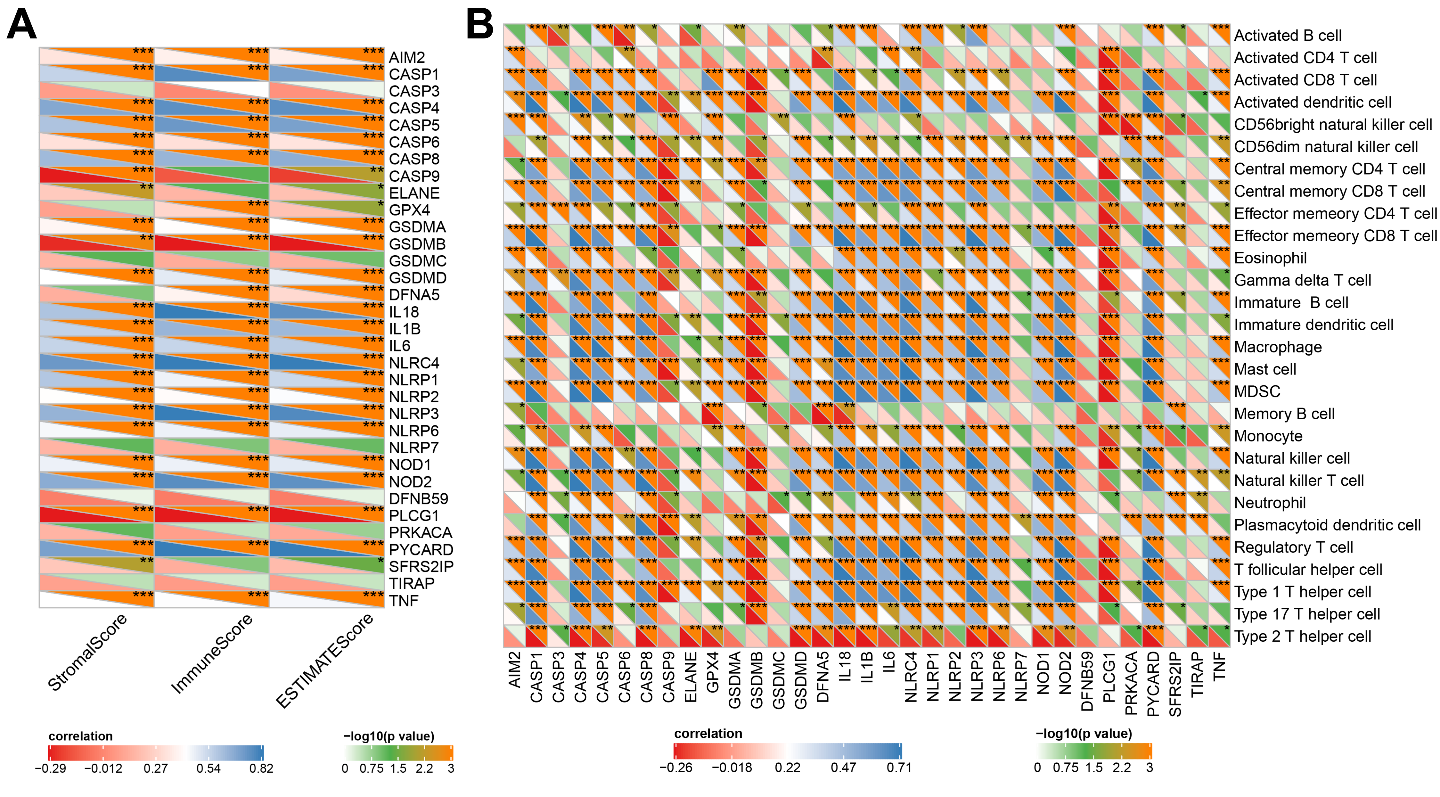


**Supplementary Figure S2.** The role of 33 pyroptosis-associated genes in the TIME of GBM. (A) The correlation of 33 pyroptosis-associated genes with stromal, immune, and ESTIMATE scores using Pearson’s analysis. (B) The correlation of 33 pyroptosis-associated genes with 28 immune cells. * p < 0.05, ** p < 0.01, *** p < 0.001. TIME, Tumor immune microenvironment; GBM, Glioblastoma


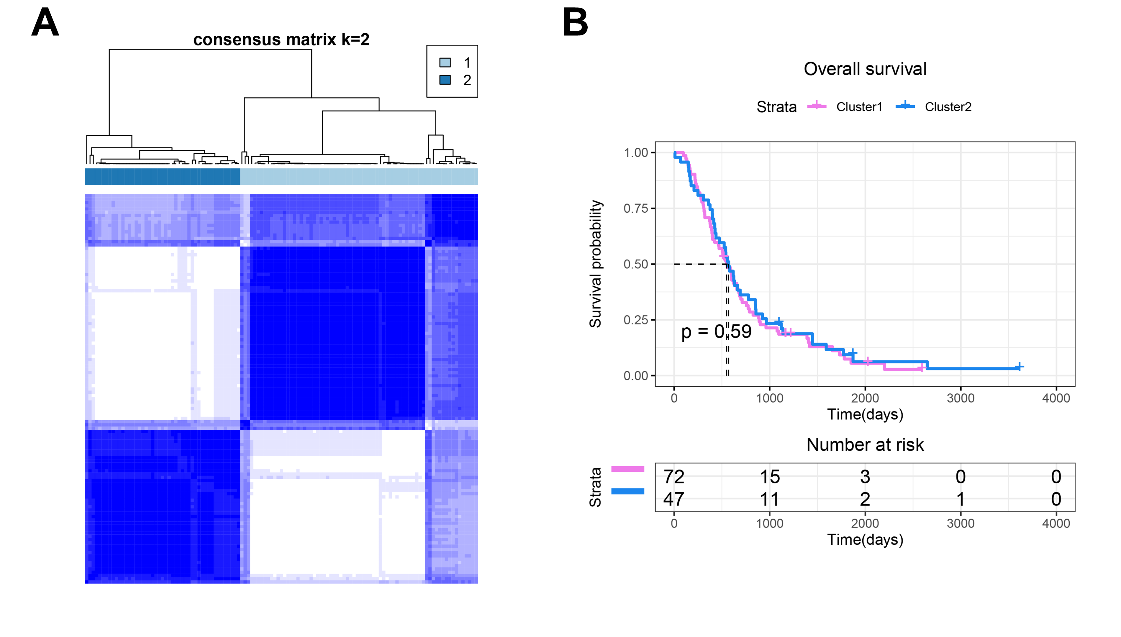


**Supplementary Figure S3.** Tumor classification based on 33 pyroptosis-associated genes in the REMBRANDT cohort. **(A)** 119 GBM patients were grouped into two clusters according to the consensus clustering matrix (k = 2). **(B)** Kaplan–Meier curve of OS for the two clusters.


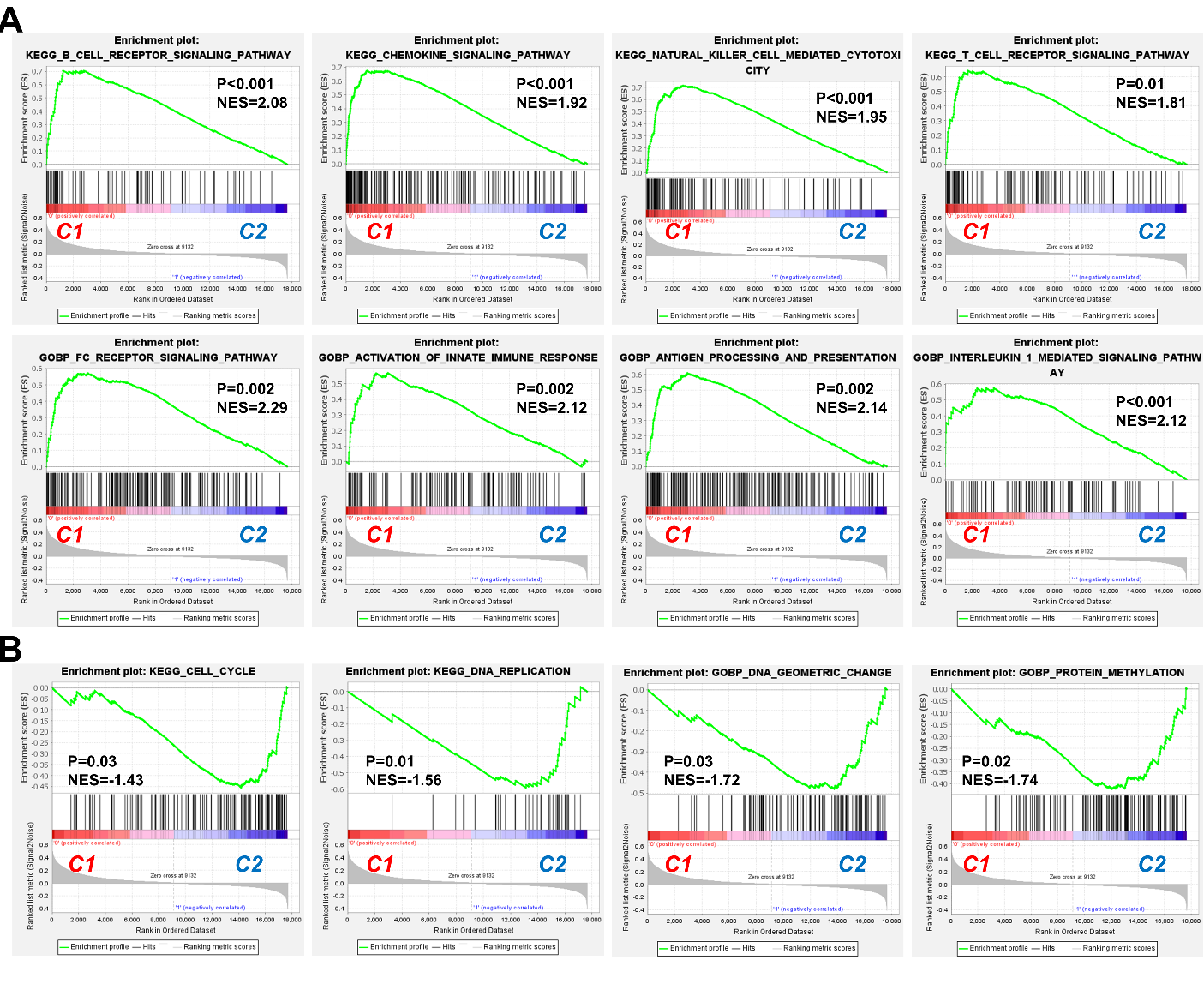


**Supplementary Figure S4.** GSEA in the TCGA cohort. **(A)** Cluster 1 enriched pathways. **(B)** Cluster 2 enriched pathways. GSEA, Gene set enrichment analysis


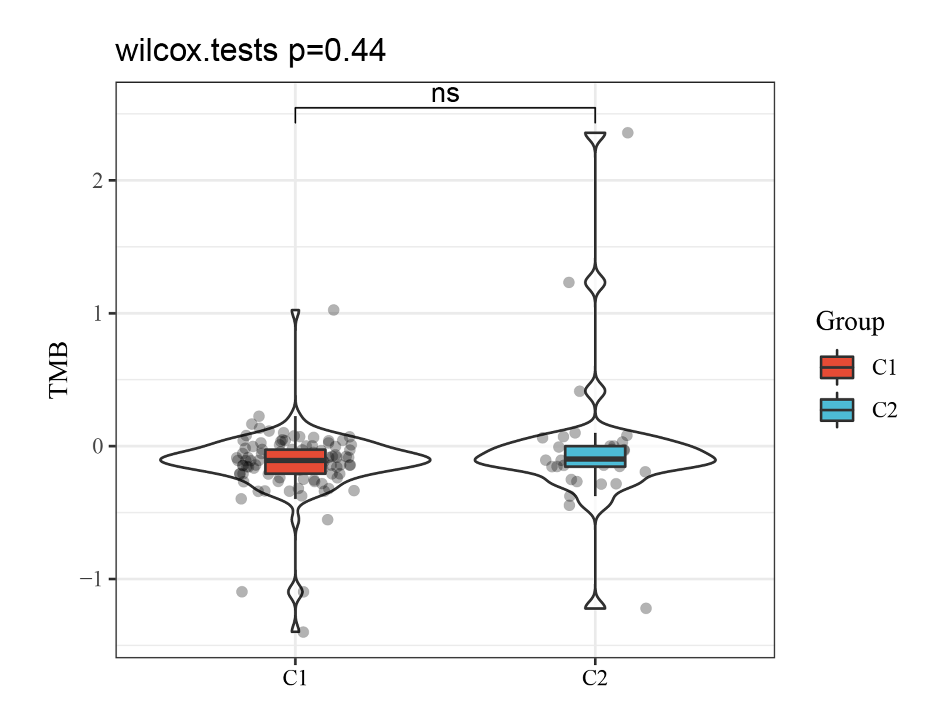


**Supplementary Figure S5.** Differences of TMB in two GBM clusters in TCGA cohort. TMB, Tumor mutation burden; GBM, Glioblastoma


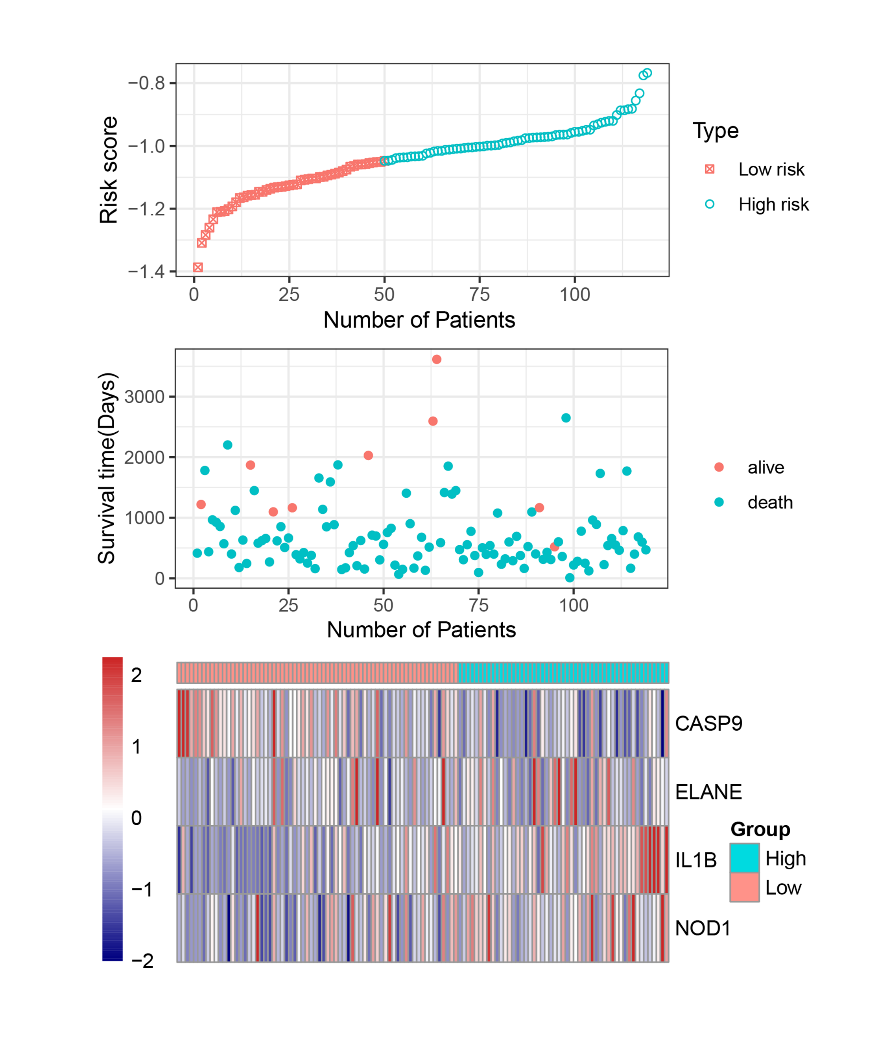


**Supplementary Figure S6.** Distribution of risk score, survival status, and the expression of four prognostic pyroptosis-associated genes in the REMBRANDT cohort.


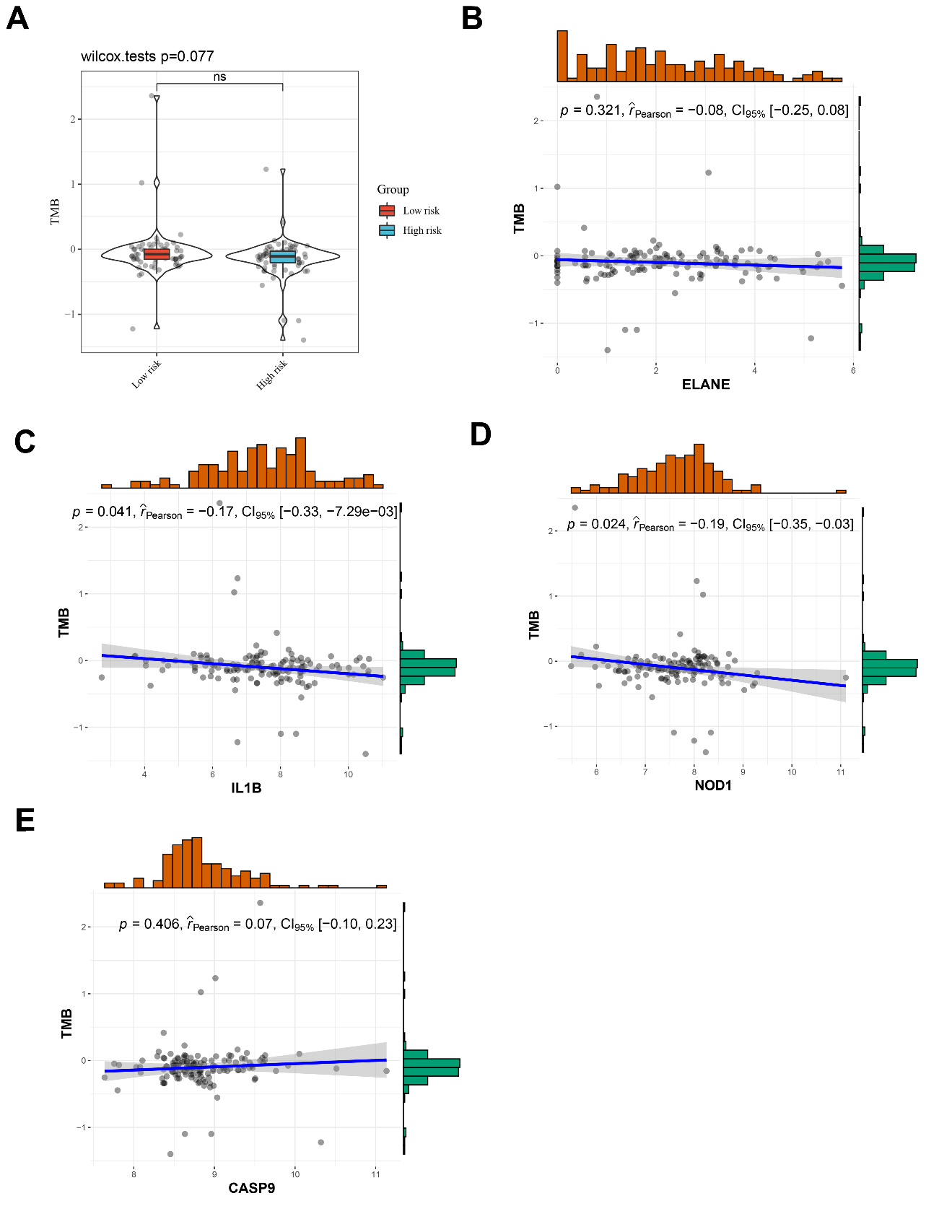


**Supplementary Figure S7.** The relationship between the risk model and TMB. **(A)** Differences in TMB between high- and low- risk groups. **(B-E)** The correlations of ELANE **(B)**, IL1B **(C)**, NOD1 **(D)**, and CASP9 **(E)** with TMB. TMB, Tumor mutation burden
