# Supplementary material for: Pyroptosis in glioblastoma: A crucial regulator of the tumour immune microenvironment and a predictor of prognosis
Source: J Cell Mol Med. 2022 Jan 26;26(5):1579–93. doi: 10.1111/jcmm.17200 (PMC8899201; doi:10.1111/jcmm.17200)
Supplement: Supplementary file 2 — Tab S1 [file JCMM-26-1579-s003.docx]

**Supplementary Table S1.** Clinical characteristics of GBM patients in the TCGA cohort and REMBRANDT cohort.

| **Characteristics** | **TCGA cohort (N=143)** | **REMBRANDT cohort (N=119)** |
| --- | --- | --- |
| **Age** |  |  |
| ≤60 years | 77 (53.85%) | 81 (68.07%) |
| >60 years | 66 (46.15%) | 38 (31.93%) |
| **Gender** |  |  |
| Male | 94 (65.73%) | 72 (60.50%) |
| Female | 49 (34.27%) | 47 (39.50%) |
| **Vital status** |  |  |
| Alive | 50 (34.97%) | 9 (7.56%) |
| Dead | 93 (65.03%) | 110 (92.44%) |
