# Supplementary material for: Pyroptosis in glioblastoma: A crucial regulator of the tumour immune microenvironment and a predictor of prognosis
Source: J Cell Mol Med. 2022 Jan 26;26(5):1579–93. doi: 10.1111/jcmm.17200 (PMC8899201; doi:10.1111/jcmm.17200)
Supplement: Supplementary file 4 — Tab S3 [file JCMM-26-1579-s005.docx]

**Supplementary Table S3.** IGP was estimated for each cluster in the validation cohort.

| Cluster | C1 | C2 |
| --- | --- | --- |
| IGP value | 0.511 | 0.950 |
